# Multiple endocrinopathies, hypercalcaemia and pancreatitis following combined immune checkpoint inhibitor use- case report and review of literature

**DOI:** 10.1186/s12902-021-00693-x

**Published:** 2021-02-27

**Authors:** Christine Newman, Oratile Kgosidalwa, Osamah A. Hakami, Carmel Kennedy, Liam Grogan, Amar Agha

**Affiliations:** 1 Department of Diabetes and Endocrinology, Beaumont Hospital, Dublin 9, Republic of Ireland; 2 Department of Medical Oncology, Beaumont Hospital, Dublin 9, Republic of Ireland

**Keywords:** Case report, Immune checkpoint inhibitor, Hypophysitis, Thyroiditis, Hypercalcaemia

## Abstract

**Background:**

Immune checkpoint inhibitors (ICIs) are a novel class of oncological agents which are used to treat a number of malignancies. To date seven agents have been approved by the Food and Drug Administration (FDA) to treat both solid and haematological malignancies. Despite their efficacy they have been associated with a number of endocrinopathies. We report a unique case of hypophysitis, thyroiditis, severe hypercalcaemia and pancreatitis following combined ICI therapy.

**Case presentation:**

A 46-year old Caucasian female with a background history of malignant melanoma and lung metastases presented to the emergency department with lethargy, nausea, palpitations and tremors. She had been started on a combination of nivolumab and ipilimumab 24 weeks earlier. Initial investigations revealed thyrotoxicosis with a thyroid stimulating hormone (TSH) of < 0.01 (0.38–5.33) mIU/L, free T4 of 66.9 (7–16) pmol/.L. TSH receptor and thyroperoxidase antibodies were negative. She was diagnosed with thyroiditis and treated with a beta blocker. Six weeks later she represented with polyuria and polydipsia. A corrected calcium of 3.54 (2.2–2.5) mmol/l and parathyroid hormone (PTH) of 9 (10–65) pg/ml confirmed a diagnosis of non-PTH mediated hypercalcaemia. PTH-related peptide and 1, 25-dihydroxycholecalciferol levels were within the normal range. Cross-sectional imaging and a bone scan out ruled bone metastases but did reveal an incidental finding of acute pancreatitis – both glucose and amylase levels were normal. The patient was treated with intravenous hydration and zoledronic acid. Assessment of the hypothalamic-pituitary-adrenal (HPA) axis uncovered adrenocorticotrophic hormone (ACTH) deficiency with a morning cortisol of 17 nmol/L. A pituitary Magnetic Resonance Image (MRI) was unremarkable. Given her excellent response to ICI therapy she remained on ipilimumab and nivolumab. On follow-up this patient’s thyrotoxicosis had resolved without anti-thyroid mediations – consistent with a diagnosis of thyroiditis secondary to nivolumab use. Calcium levels normalised rapidly and remained normal. ACTH deficiency persisted, and she is maintained on oral prednisolone.

**Conclusion:**

This is a remarkable case in which ACTH deficiency due to hypophysitis; thyroiditis; hypercalcaemia and pancreatitis developed in the same patient on ipilimumab and nivolumab combination therapy. We postulate that hypercalcaemia in this case was secondary to a combination of hyperthyroidism and secondary adrenal insufficiency.

## Background

Immune check point inhibitors (ICIs) are a recently established group of oncology medications which are effective in the treatment of aggressive malignancies. To date the FDA has approved seven such medications. Despite considerable anti-tumour effect, they can cause multi-systemic adverse events (AEs). Endocrinopathies account for 10% of all AEs associated with ICIs [1] and occur in up to 18% of patients. Moreover, endocrinopathies are more common when two ICIs are used together [2]. The thyroid and pituitary glands are the most commonly affected endocrine organs [3], however cases of type 1 diabetes mellitus (including diabetic ketoacidosis) [4], primary adrenal insufficiency [5], Cushing’s Syndrome [6], diabetes insipidus [7] and hypoparathyroidism [8] have all been reported. Patients can develop problems with one or multiple endocrine organs. Such problems can arise simultaneously or in sequence [9].

While many non-endocrine side effects are severe enough to merit cessation of ICI therapy, the majority of endocrine related adverse events can be adequately managed and treatment with ICIs can be continued. Treatment for endocrine immune-related adverse events (irAEs) is usually lifelong.

We present the first case of a 46 year old woman who developed multiple endocrinopathies, hypercalcaemia and pancreatitis when exposed to ipilimumab and nivolumab for the treatment of metastatic melanoma. Each of these conditions was successfully treated and the patient was able to continue ICI therapy.

## Case presentation

A 46 year old lady presented to the emergency department with a 1 week history of lethargy, nausea, palpitations, and tremors. She had recently received her eighth cycle of ipilimumab and nivolumab for malignant melanoma with lung metastases.

She had experienced multiple minor adverse reactions earlier in her therapy (including gastrointestinal disturbances and headaches), however the size and number of lung metastases had reduced and ICI treatment was continued.

Her initial general physical examination was unremarkable and biochemical tests including a full blood count, renal and liver profiles were within normal limits. A cranial MRI out ruled brain metastases or leptomeningeal disease.

Thyroid function tests (TFTs) showed biochemical thyrotoxicosis with an elevated free T4 of 66.9 (7–16) pmol/L and a TSH level of < 0.01 (0.38–5.33) mIU/L, with negative TRAB and TPO antibodies. The TFT results together with recent nivolumab use supported a diagnosis of thyroiditis. This was confirmed by a pertechnetate technetium uptake scan which showed reduced intensity of tracer uptake in the thyroid gland.

**Fig. 1:**
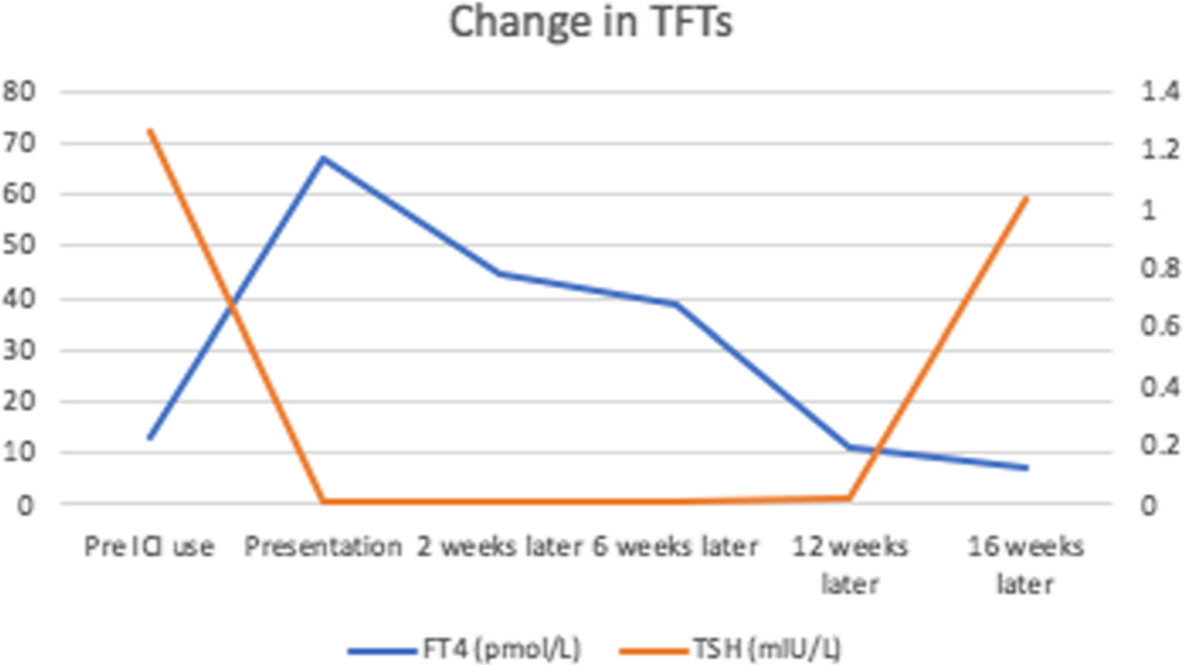
Thyroid function tests (TFTs) before and during Immune Checkpoint Inhibitor (ICI) use

Treatment with beta blockade was commenced and the patient showed symptomatic improvement. Serial TFTs also improved (Fig. 1) and ICI therapy was continued.

Six weeks after the diagnosis of thyroiditis, the patient presented with polydipsia and polyuria. Biochemical investigations showed a corrected calcium of 3.54 (2.15–2.5) mmol/L and normal glucose, phosphate and magnesium levels. PTH levels were suppressed at 9 (15–65) pg/ml. Although 25 hydroxyvitamin D level was insufficient at 35.8 nmol/L (> 50 nmol/L considered sufficient) the active form 1,25 dihyroxyvitamin D level was within normal limits. This indicated that hypercalcaemia was not due to hyperparathyroidism or hypervitaminosis D. PTH-related protein level was also normal.

**Fig. 2:**
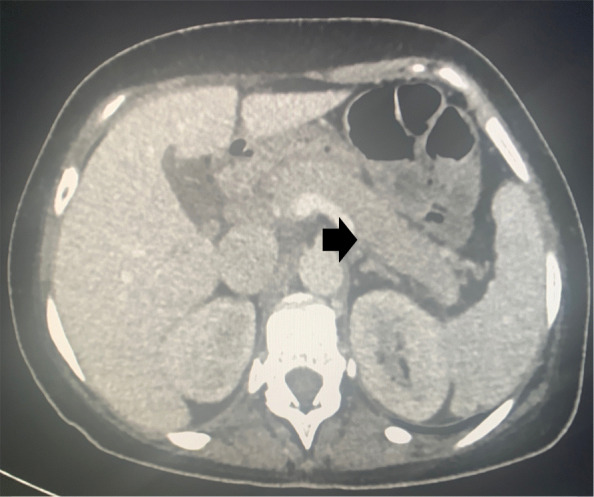
CT Abdomen*.* A CT image of the abdomen*.* The pancreas appears diffusely oedematous (particularly noticeable in the body and tail [black arrow]) with very mild surrounding inflammatory stranding

Although a computerised tomography (CT) scan of thorax, abdomen and pelvis and skeletal scintigraphy did not show any evidence of bone metastasis, the CT demonstrated radiological evidence of acute pancreatitis (Fig. 2). The patient denied any significant abdominal pain and both amylase and glucose levels were normal.

In this instance hypercalcaemia was successfully and quickly treated with intra-venous fluids and Zoledronic acid and on day 2 of admission the patient’s corrected calcium was 2.1 mmol/L.

The patient continued to experience significant fatigue and was investigated for adrenal insufficiency. Her morning cortisol level returned at 17 (185–624) nmol/L; she also had a suppressed ACTH level of < 3 (7.2–62.4) pg/ml, a normal Renin level of 18.7 mIU/L (6.1–62.7 mIU/L) and negative anti-adrenal antibodies. In the absence of previous glucocorticoid use this was suggestive of secondary (central) adrenal insufficiency. The patient reported regular menses and a dedicated pituitary MRI showed a normal pituitary gland and stalk.

Based on these results the patient was diagnosed with isolated ACTH deficiency due to ipilimumab induced hypophysitis. We proposed that a combination of adrenal insufficiency and profound thyroiditis resulted in hypercalcaemia.

The patient was commenced on oral hydrocortisone replacement and received comprehensive education regarding the sick day rules.

Eight weeks later (14 weeks after her first presentation with thyroiditis) a second short synacthen test was performed to assess HPA axis recovery., The 30 min cortisol value was 38 nmol/L (normal value > 450 nmol/L) indicating ongoing adrenal insufficiency.

More than 1 year on from her initial thyroiditis the patient is clinically and biochemically euthyroid with a corrected calcium of 2.39 mmol/L. She continues to take oral glucocorticoids - she was changed from oral hydrocortisone to oral prednisolone following a diagnosis of seronegative arthritis). Her most recent radiological investigations show an excellent response to ICI therapy and she is followed by the dermatology, oncology and endocrinology services.

## Discussion and conclusions

We present the case of multiple endocrinopathies, non-PTH mediated hypercalcaemia and pancreatitis following combined ipilimumab and nivolumab therapy.

Both of these medications belong to the class of drugs known as immune checkpoint inhibitors (ICIs) which target immune checkpoint regulator proteins. Recent research has focused on 4 particular targets namely

- ○ Cytotoxic T-lymphocyte associated antigen 4 (CTLA-4)
- ○ Programmed cell death protein-1 (PD-1) also found on lymphocytes and
- ○ Programmed death ligand 1 and 2 (PDL-1 and PDL-2) found on multiple cells

In their normal state immune checkpoint regulator proteins reduce the immune response to antigenic or tumour cells which allows tumour cells to proliferate [10]. ICIs inhibit these immune regulator proteins and allow the activation of T lymphocyte which then successfully target and eliminate tumour cells.

To date seven ICIs have FDA approval for a number of malignancies including breast, hepatocellular, renal, gastric, lung and haematological malignancies. They are also commonly used to treat malignant melanoma.

Despite their frequent use, ICIs have been associated with multi-systemic immune-related adverse events (irAEs) due to uninhibited T cell activation. Up to 10% of irAEs are endocrine related [1]. Endocrinopathies are usually permanent [11], however they can be effectively treated and ICIs can usually be continued.

Ipilimumab, a CTLA-4 inhibitor is known to cause hypophysitis. Hypophysitis has an incidence of 1 per 9 million in the general population [12] however with ipilimumab use, rates of hypophysitis vary between 3.2% [13] and 17% [14]. The rate of hypophysitis increases further when ipilimumab and nivolumab (a PD-1 inhibitor) are combined [15].

Symptoms of hypophysitis typically occur after 12 weeks of treatment and can be non-specific. Patients frequently list headache and fatigue as their initial symptoms and a high degree of clinical suspicion is essential. Adrenal, thyroid and gonadotrophin axes are the most commonly affected however multiple hormonal dysfunctions can occur together [16]. The pituitary gland in commonly affected as is expresses a high number of CTLA-4 proteins [17].

Pituitary imaging with MRI typically reveals mild to moderate pituitary enlargement, with or without stalk thickening. Involvement of the optic chiasm is rarely observed [18] and MRI is normal in up to a third of patients [19].

Though individual case reports of HPA axis recovery have been published [20], ACTH deficiency is usually irreversible [21]. Conversely gonadotrophins and thyroid axes display some recovery [18].

Thyroid dysfunction is the most common irAE seen in ICs use and has occurs in 35% of cases [22]. Like hypophysitis, a combination of CTLA-4 and PD-1 use increases the risk of developing thyroid dysfunction [23]. ICI use can result in a broad spectrum of thyroid disease. Patients can develop thyroiditis, rarely Graves’ disease (occasionally severe enough to induce thyroid storm [24]) or hypothyroidism. Thyroid dysfunction is also more common in those with a history of pre-existing thyroid disease [1]. Hypothyroidism can occur as a first presentation of thyroid dysfunction or as a consequence of thyroiditis [9]. Corticosteroids or beta-blockers can be used to treat hyperthyroidism and anti-thyroid drugs are rarely needed. Thyroid dysfunction typically occurs early in treatment (average of 6 weeks) [23] and ICI therapy can normally be continued once symptoms are controlled.

Hypercalcaemia has also been described following ICI use [25, 26]. In this case we postulated that the combination of hyperthyroidism and adrenal insufficiency contributed to hypercalcaemia. In a case report by Takebayashi et al. [25] showed similarities to our case, whereby a patient developed hypercalcaemia which also responded very quickly to bisphosphonate therapy and rehydration. However, in our patient we also note that these treatment strategies coincided with steroid replacement and treatment of hyperthyroidism which may have helped reduce calcium levels. It is also possible that hypercalcaemia was caused by ICI therapy itself, however the patient’s calcium normalised despite ongoing exposure to ICIs.

Finally, the patient was also diagnosed with pancreatitis on imaging. Pancreatitis is seen in < 1% of patients on ICI therapy [27]. Symptoms of pancreatitis include abdominal pain and hyperglycaemia. Patients may also be asymptomatic. Pancreatitis usually develops within 20 weeks of therapy. Though pancreatitis has been described in association with hypophysitis [26] we believe that this is the first case report to describe hypophysitis, thyroiditis, non-PTH medicated hypercalcaemia (in the absence of bone metastases) and pancreatitis in the same patient.

In conclusion, this is a complex case of multiple endocrinopathies associated with ICI use in a patient with metastatic malignant melanoma. This case highlights the range of irAEs seen in ICI use and the need for a high index of suspicion for endocrine dysfunction when managing these patients. It also demonstrates how with prompt diagnosis and appropriate management patients can continue on this effective therapy.

## Data Availability

Not applicable.

## Abbreviations

ACTH: adrenocorticotrophic hormone
AEs: Adverse events
CT: computed tomography
CTLA-4: Cytotoxic T-lymphocyte associated antigen 4
FDA: Food and Drug Administration
HPA: Hypothalamic-pituitary-adrenal axis
ICI: Immune check point inhibitors
irAEs: immune-related adverse events
MRI: Magnetic Resonance Imagine
PD-1: Programmed cell death protein-1
PDL: Programmed death ligand
PTH: Parathyroid Hormone
TFTs: Thyroid function tests
TPO: thyroperoxidase
TRAB: TSH receptor autoantibodies
TSH: Thyroid Stimulating Hormone

## References

[1] Ronan K, Othman EHS, McKenna S, Anderson C, Sheehan D, Griffin M (2019). Immunotherapy-induced endocrinopathies: a multicentre experience. J Clin Oncol.

[2] Scott ES, Long GV, Guminski A, Clifton-Bligh RJ, Menzies AM, Tsang VH (2018). The spectrum, incidence, kinetics and management of endocrinopathies with immune checkpoint inhibitors for metastatic melanoma. Eur J Endocrinol.

[3] Kassi E, Angelousi A, Asonitis N, Diamantopoulos P, Anastasopoulo A, Papaxoinis G (2019). Endocrine-related adverse events associated with immune-checkpoint inhibitors in patients with melanoma. Cancer Med.

[4] Clotman K, Janssens K, Specenier P, Weets I, De Block CEM (2018). Programmed cell death-1 inhibitor-induced type 1 diabetes mellitus. J Clin Endocrinol Metab.

[5] Paepegaey AC, Lheure C, Ratour C, Lethielleux G, Clerc J, Bertherat J (2017). Polyendocrinopathy resulting from pembrolizumab in a patient with a malignant melanoma. J Endocr Soc.

[6] Lupu K, Pages C, Laly P, Deylon J, Laloi M, Petit A (2017). Transient pituitary ACTH-dependent Cushing syndrome caused by an immune checkpoint inhibitor combination. Melanoma Reg.

[7] Zhao C, Tella SH, Del Rivero J, Kommalapati A, Ebenuwa I, Gulley JL (2018). Anti-PD-L1 treatment induced central diabetes insipidus. J Clin Endocrinol Metab.

[8] Piranavan P, Li Y, Brown E, Kemp EH, Trivedi N (2019). Immune checkpoint inhibitor-induced hypoparathyroidism associated with calcium-sensing receptor-activating autoantibodies. J Clin Endocrinol Metab.

[9] Tan MH, Iyengar R, Mizokami-Stout K, Yentz S, MacEachern MP, Shen LY, et al. Spectrum of immune checkpoint inhibitors-induced endocrinopathies in cancer patients: a scoping review of case reports. Clin Diabetes Endocrinol. 2019. 10.1186/s40842-018-0073-4. 10.1186/s40842-018-0073-4 PMC6343255 30693099

[10] Darvin P, Toor SM, Nair VS, Elkord E (2018). Immune checkpoint inhibitors: recent progress and potential biomarkers. Exp Mol Med.

[11] McGowan A, Weatherby T, Powlson A, Parkinson C, Chatterjee K, Corrie P, et al. Endocrinopathies are a frequent consequence of immune-checkpoint inhibitor therapy, with a low recover rate of both thyroid and pituitary dysfunction. Endocr Abstr. 2017. 10.1530/endoabs.50.P251.

[12] Caturegli P, Newschaffer C, Olivi A, Pomper MG, Burger PC, Rose NR (2005). Autoimmune hypophysitis. Endocr Rev.

[13] Albarel F, Gaudy C, Castinetti F, Carre T, Morange I, Conte-Devolx B (2015). Long term follow-up of ipilimumab-induced hypophysitis, a common adverse event of the anti-CTLA-4 antibody in melanoma. Eur J Endocrinol.

[14] Maker AV, Yang JC, Sherry RM, Topalian SL, Kammula US, Royal RE (2006). Intrapatient dose escalation of anti-CTLA-4 antibody in patients with metastatic melanoma. J Immunother.

[15] Barroso-Sousa R, Barry WT, Garrido-Castro AC, Hodi FS, Min L, Krop IE, Tolaney SM (2018). Incidence of endocrine dysfunction following the use of different immune checkpoint inhibitor regimens: a systematic review and meta-analysis. JAMA Oncol.

[16] Levy M, Abeillon J, Borson-Chazot F, Daile S, Raverot G, Anceau CC. Immune checkpoint inhibitors therapy-induced hypophysitis is frequently associated with previous thyroid disorders: results from ImmuCare study. Endocr Abstr. 2019. 10.1530/endoabs.63.GP238.

[17] Caturegli P, Di Dalmazi G, Lombardi M, Grosso F, Laman T, Taverna G (2016). Hypophysitis secondary to cytotoxic T-lymphocyte-associated protein 4 blockade: insights into pathogenesis from an autopsy series. Am J Pathol.

[18] Faje AT, Sullivan R, Lawrence D, Tritos NA, Fadden R, Klibanski A (2014). Ipilimumab-induced hypophysitis: a detailed longitudinal analysis in a large cohort of patients with metastatic melanoma. J Clin Endocrinol Metab.

[19] Mahzari M, Liu D, Arnaout A, Lochnan H (2015). Immune checkpoint inhibitor therapy associated hypophysitis. Clin Med Insights Endocrinol Diabetes.

[20] Thapi S, Leiter A, Galsky M, Gallagher EJ (2019). Recovery from secondary adrenal insufficiency in a patient with immune checkpoint inhibitor therapy induced hypophysitis. J Immunother Cancer.

[21] Girotra M, Hansen A, Farooki A, Byun DJ, Min L, Creelan BC, et al. The current understanding of the endocrine effects from immune checkpoint inhibitors and recommendations for management. JNCI Cancer Spectr. 2018. 10.1093/jncics/pky021. 10.1093/jncics/pky021 PMC6054022 30057972

[22] Patel NS, Oury A, Daniels GA, Bazhenova L, Patel SP (2018). Incidence of thyroid function test abnormalities in patients receiving immune-checkpoint inhibitors for cancer treatment. Oncologist..

[23] Iyer PC, Cabanillas ME, Waguespack SG, Hu MI, Thosani S, Lavis VR, et al. Immune-related thyroiditis with checkpoint inhibitors. Thyroid. 2018. 10.1089/thy.2018.0116. 10.1089/thy.2018.0116 PMC6157359 30132401

[24] McMillen B, Dhillon MS, Yong-Yow S. A rare case of thyroid storm. BMJ Case Rep. 2016. 10.1136/bcr-2016-214603. 10.1136/bcr-2016-214603 PMC4840694 27090545

[25] Takebayashi K, Ujiie A, Kubo M, Furukawa S, Yamauchi M, Shinozaki H (2018). Isolated adrenocorticotropic hormone deficiency and severe hypercalcemia after destructive thyroiditis in a patient on nivolumab therapy with a malignant melanoma. J Clin Med Res.

[26] Ryder M, Callahan M, Postow MA, Wolchok J, Fagin JA (2014). Endocrine-related adverse events following ipilimumab in patients with advanced melanoma: a comprehensive retrospective review from a single institution. Endocr Relat Cancer.

[27] Tirumani SH, Ramaiya NH, Keraliya A, Bailey ND, Ott PA, Hodi FS (2015). Radiographic profiling of immune-related adverse events in advanced melanoma patients treated with ipilimumab. Cancer Immunol Res.

